# Transformer-CNN: Swiss knife for QSAR modeling and interpretation

**DOI:** 10.1186/s13321-020-00423-w

**Published:** 2020-03-18

**Authors:** Pavel Karpov, Guillaume Godin, Igor V. Tetko

**Affiliations:** 1 Institute of Structural Biology, Helmholtz Zentrum München-Research Center for Environmental Health (GmbH), Ingolstädter Landstraße 1, 85764 Neuherberg, Germany; 2 BIGCHEM GmbH, Ingolstädter Landstraße 1, 85764 Neuherberg, Germany; 3 Firmenich International SA, Digital Lab, Geneva, Lausanne, Switzerland

**Keywords:** Transformer model, Convolutional neural neural networks, Augmentation, QSAR, SMILES, Embeddings, Character-based models, Cheminformatics, Regression, Classification

## Abstract

We present SMILES-embeddings derived from the internal encoder state of a Transformer [1] model trained to canonize SMILES as a Seq2Seq problem. Using a CharNN [2] architecture upon the embeddings results in higher quality interpretable QSAR/QSPR models on diverse benchmark datasets including regression and classification tasks. The proposed Transformer-CNN method uses SMILES augmentation for training and inference, and thus the prognosis is based on an internal consensus. That both the augmentation and transfer learning are based on embeddings allows the method to provide good results for small datasets. We discuss the reasons for such effectiveness and draft future directions for the development of the method. The source code and the embeddings needed to train a QSAR model are available on https://github.com/bigchem/transformer-cnn. The repository also has a standalone program for QSAR prognosis which calculates individual atoms contributions, thus interpreting the model’s result. OCHEM [3] environment (https://ochem.eu) hosts the on-line implementation of the method proposed.

## Introduction

Quantitative Structure–Activity (Property) Relationship (QSAR/QSPR) approaches find a nonlinear function, often modelled as an artificial neural network (ANN), that estimates the activity/property based on a chemical structure. In the past, most QSAR works heavily relied on descriptors [4] that represent in a numerical way some features of a complex graph structure of a compound. Amongst numerous families of descriptors, the fragment descriptors that count occurrences of a subgraph in a molecule graph, hold a distinctive status due to simplicity in the calculation. Moreover, there is a theoretical proof that one can successfully build any QSAR model with them [5]. Even a small database of compounds contains thousands of fragmental descriptors and some feature selection algorithm has traditionally been used to find a proper subset of descriptors for better quality, and to speed up the whole modeling process. Thus, feature selection in conjunction with a suitable machine learning method was key to success [6]. The rise of deep learning [7] allows us to bypass tiresome expert and domain-wise feature construction by delegating this task to a neural network that can extract the most valuable traits of the raw input data required for modeling the problem at hand [8, 9].

**Fig. 1:**
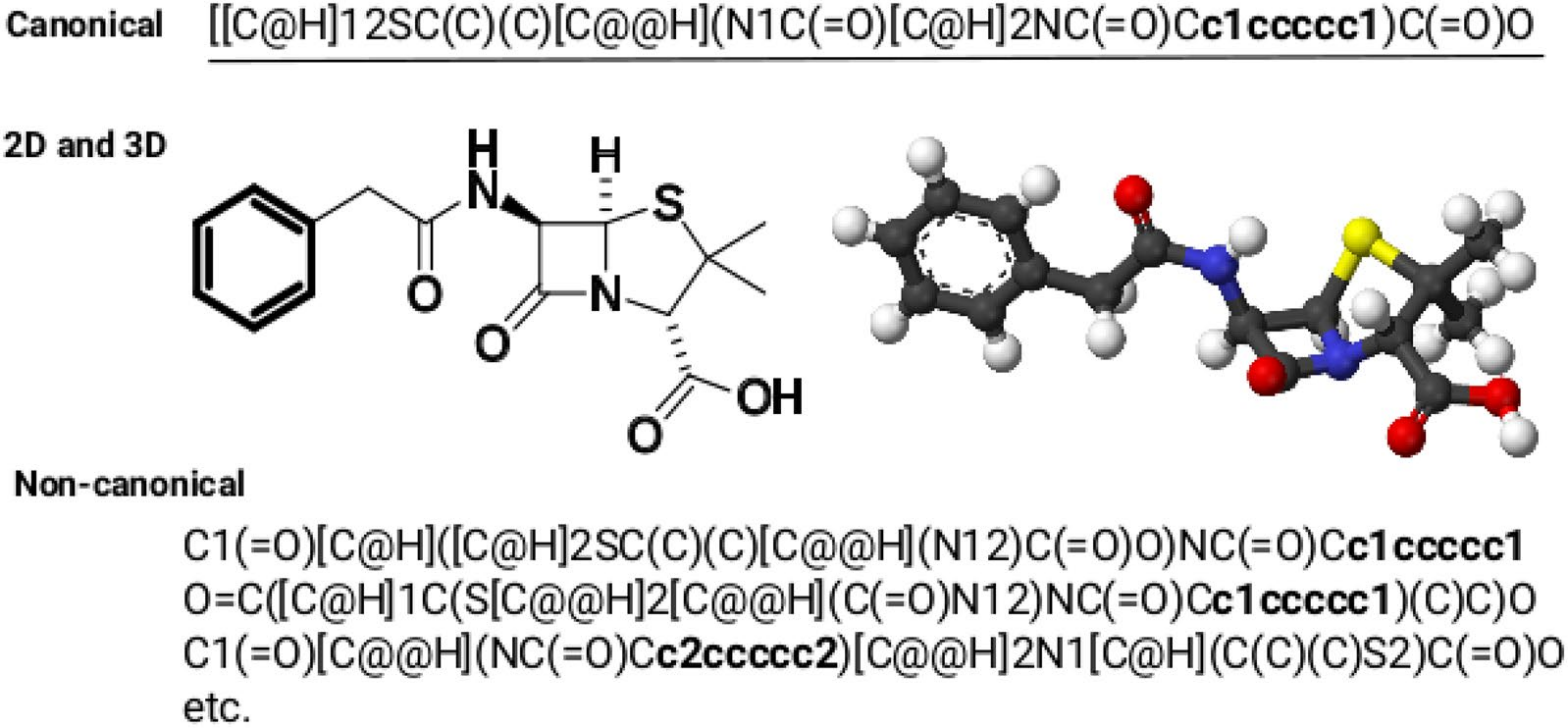
Benzylpenicillin canonical SMILES at the top, 2D and 3D structures derived from SMILES with OpenBabel [15] in the middle, and three non-canonical SMILES examples at the bottom. A substructure of the phenyl ring is written in bold font

In this setting, the whole molecule as a SMILES-strings [10, 11] (Simplified Molecular Input Line Entry System) or a graph [12, 13] serves as the input to the neural network. SMILES notation allows for the writing of any complex formula of an organic compound in a string facilitating storage and retrieval information about molecules in databases [14]. It contains all information about the compound sufficient to derive the entire configuration (3D-structure) and has a direct connection to the nature of fragmental descriptors, Fig. 1, thus, making SMILES one of the best representation for QSAR studies.

One of the first works exploiting direct SMILES input as descriptors used fragmentation of strings into groups of overlapping substrings forming a SMILES-like set or a hologram of a molecule [16]. Within this approach, there was no need to derive a 2D/3D configuration of the molecule with subsequent calculation of descriptors keeping the quality of the models at the same level as with classical descriptors or even better.

SMILES strings are sequences of characters; therefore, they can be analyzed by machine-learning methods suitable for text processing, namely with convolutional and recurrent neural networks. After the demonstration of text understanding from character-level inputs [17], this technique was adopted in chemoinformatics [11, 18–21]. Recently, we showed that the augmentation of SMILES (using canonical as well as non-canonical SMILES during model training and inference) increases the performance of convolutional models for regression and classification tasks [22].

**Fig. 2:**
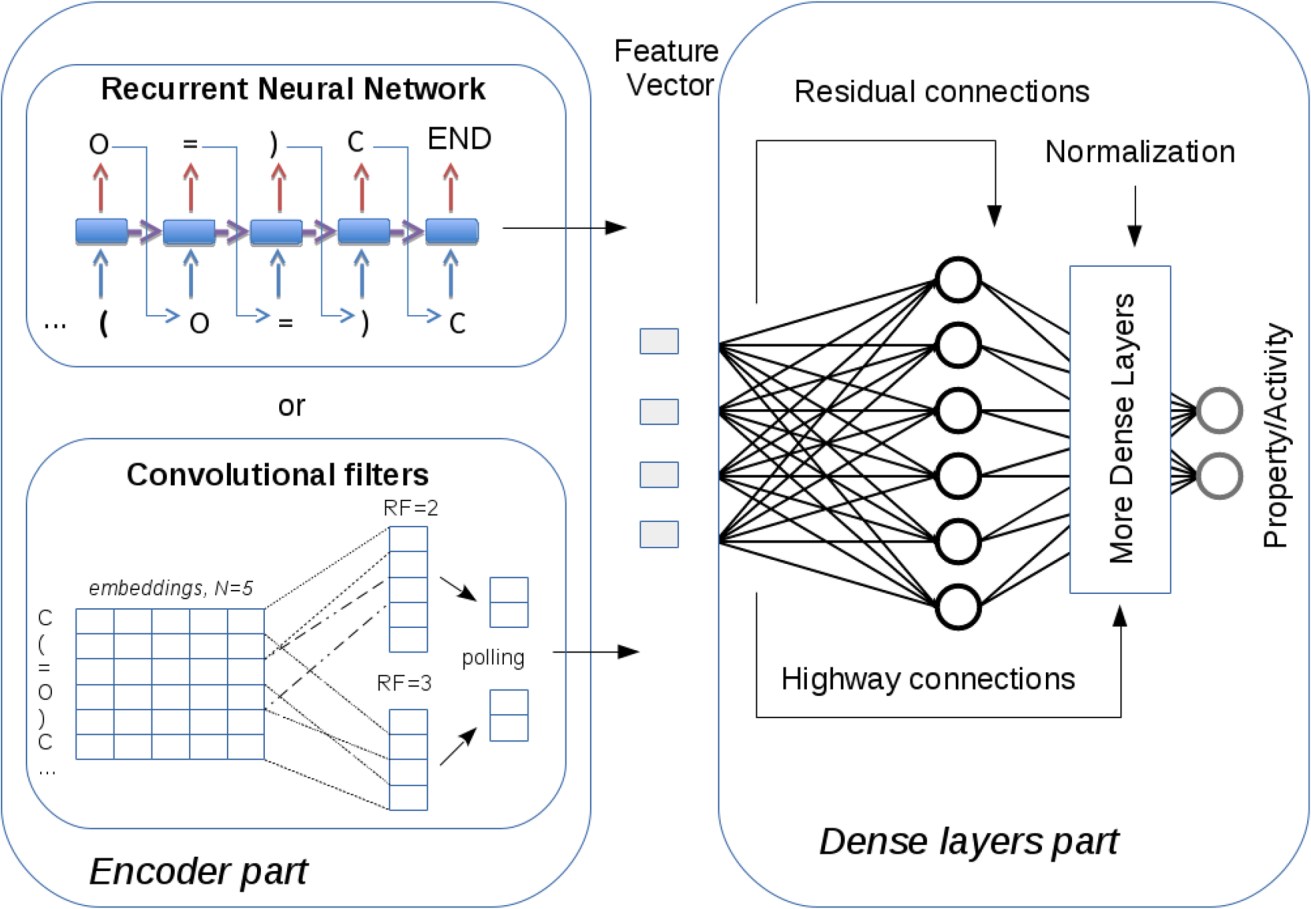
Scheme of modern QSAR models based on ANN. The encoder part (left) extracts main features of the input data by means of RNN (top) or convolutional layers (bottom). Then the feature vector as usual descriptors feeds to the dense layer part consisting of residual and highway connections, normalization layers, and dropouts

Technically modern machine-learning models consist of two parts working together. The first part encodes the input data and extracts the most robust features by applying convolutional filters with different receptive fields (RF) or recurrent layers, whereas the second part directly builds the regular model based on these features using standard dense layers as building blocks (so called classical “MLP”), Fig. 2. Though powerful convolutional layers can effectively encode the input within its internal representation, usually one needs a considerable training dataset and computational resources to train the encoder part of a network.

The concept of embeddings mitigates the problem by using the pre-trained weights designed for image [23] or text processing [24] tasks. It allows transfer learning from previous data and speeds up the training process for building models with significantly smaller datasets inaccessible for training from scratch. Typically, QSAR datasets contain only several hundreds of molecules, and SMILES-embeddings could improve models by developing better features.

One way of separately obtaining SMILES embeddings is to use classical autoencoder [25] approach where the input is the same as the output. In the case of SMILES, however, it would be more desirable to explore a variety of SMILES belonging to the same molecule due to redundant SMILES grammar, Fig. 1. We hypothesized that it is possible to train a neural network to conduct a SMILES canonicalization task in a Sequence-to-Sequence (Seq2Seq) manner like a machine translation problem, where on the left side are non-canonical SMILES, and on the right side are their canonical equivalents. Recently, Seq2Seq was successfully applied to translation from InChi [26] codes to SMILES (Inchi2Sml) as well as from SMILES arbitrary to canonical SMILES (Sml2canSml), and to build QSAR models on extracted latent variables [27].

The state-of-the-art neural architecture for machine translation consists of stacked Long Short-Term Memory (LSTM) cells [28]. The training process for such networks inherently has all kinds of Recurrent Neural Networks difficulties, e.g., vanishing gradients, and the impossibility of parallelization. Recently, a Transformer model [1] was proposed where all recurrent units are replaced with convolutional and element-wise feed-forward layers. The whole architecture shows a significant speed-up during training and inference with improved accuracy over translation benchmarks. The Transformer model was applied for prediction of reaction outcomes [29] and for retrosynthesis [30].

Modern machine learning architectures although demonstrating incredible performance still lack interpretability. Explaining the reasons for a particular prediction of a model avoids “Clever Hans” predictors with spurious or non-relevant correlations [31] and foster trust and verifiability. One of the promising methods to open a “black box” uses the Layer-wise Relevance Propagation (LRP) algorithm [32], which splits the overall predicted value to a sum of contributions of individual neurons. In this method, the sum of relevance of all neurons of a layer, including the bias neuron, is kept constant. Propagation of the relevance from the last layer to the input layer allows the evaluation of the contributions of particular input features in to select the most relevant features for the whole training set [33] or to explain the individual neural network prediction [32]. We apply the LRP method for an explanation of individual results, checking the model get results for the right reason.

Our contributions in the article are as follows:

- Presenting a concept of dynamic SMILES embeddings that may be useful for a wide range of cheminformatics tasks;
- Scrutinizing CharNN models based on these embeddings for regression and classification tasks and show that the method outperforms the state-of-the-art models;
- Interpretation of the model based on LRP method;
- Our implementation as well as source codes and SMILES-embeddings are available on https://github.com/bigchem/transformer-cnn. We also provide ready-to-use implementation on https://ochem.eu within the OCHEM [3] environment and a standalone program for calculating properties and explaining the results.

## Methods

### SMILES canonicalization model

#### Dataset

**Fig. 3:**
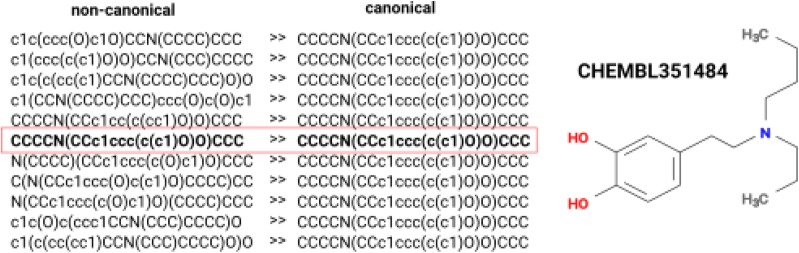
Example of the data in the training file for canonicalization model of a small molecule CHEMBL351484. Every line contains a pair of non-canonical (left) and canonical (right) separated by “ >> ”. One line has identical SMILES on both sides, stressed with the red box

To train the ANN to perform SMILES canonicalization, we used the ChEMBL database [34] with SMILES strings of length less than or equal 110 characters (> 93% of the entire database). The original dataset was augmented 10 times up to 17,657,995 canonicalization pairs written in reactions format separated by ‘ >> ’. Each pair contained on the left side a non-canonical, and on the right side—a canonical SMILES for the same molecule. Such an arrangement of the training dataset allowed us to re-use the previous Transformer code, which was originally applied for retrosynthetic tasks [30]. For completeness, we added for every compound a line where both left and right sides were identical, i.e. canonical SMILES, Fig. 3. Thus each molecule was present in the training set 11 times. If a molecule had tautomeric forms then all of them were accounted for as separate entries in the training data file.

#### Model input

Seq2Seq models use one-hot encoding vector for the input. Its values are zero everywhere except the position of the current token which is set to one. Many works on SMILES use tokenization procedure [35, 36] that combines some characters, for example ‘B’ and ‘r’ to one token ‘Br’. Other rules for handling most common two-letters elements, charges, and stereochemistry also are used for preparing the input for the neural network. According to our experience, the use of more complicated schemes instead of simple character-level tokenization did not increase the accuracy of models [30]. Therefore a simple character-level tokenization was used in this study. The vocabulary of our model consisted of all possible characters from ChEMBL dataset and has 66 symbols: ^#%()+–./0123456789=@ABCDEFGHIKLMNOPRSTVXYZ[\]abcdefgilmnoprstuy$

Thus, the model could handle the entire diversity of drug-like compounds including stereochemistry, different charges, and inorganic ions. Two special characters were added to the vocabulary: ‘^’ to indicate the start of the sequence, and ‘$’ to inform the model of the end of data input.

#### Transformer model

The canonicalization model used in this work was based upon a Transformer architecture consisting of two separate stacks of layers for the encoder and the decoder, respectively. Each layer incorporated some portion of knowledge written in its internal memory (V) with indexed access by keys (K). When new data arrived (Q), the layer calculated attention and modified the input accordingly (see the original work on Transformers [1]), thus, forming the output of the self-attention layer and weighting those parts that carry the essential information. Besides a self-attention mechanism, the layer also contained several position-wise dense layers, a normalization layer, and residual connections [1, 37]. Our model utilized a three layer architecture of Transformer with 10 blocks of self-attention, i.e. the same one as used in our previous study [30]. After the encoding process was finished, the output of the top encoder layer contained a representation of a molecule suitable for decoding into canonical SMILES. In this study we used this representation as a well-prepared latent representation for QSAR modeling.

Tensorflow v1.12.02 [38] was used as machine-learning framework to develop all parts of the Transformer, whereas RDKit v.2018.09.2 [39] was used for SMILES canonicalization and augmentation.

#### QSAR model

**Fig. 4:**
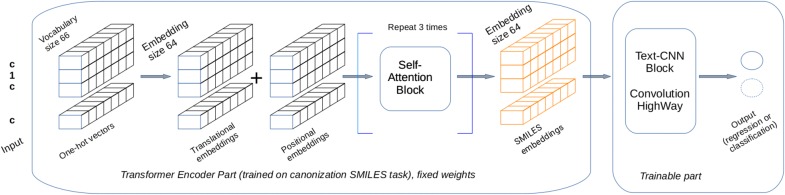
The architecture of the Transformer-CNN network

We call the output of the Transformer's encoder part a dynamic SMILES-embedding, Fig. 4. For a molecule with *N*-characters, the encoder produces the matrix with dimensions (*N*, *EMBEDDINGS*). Though technically this matrix is not an embedding because equivalent characters have different values depending on position and surroundings, it can be considered so due to its role: to convert an input one-hot raw vectors to real-value vectors in some latent space. Because these embeddings have variable lengths, we used a series of 1D convolutional filters as implemented in DeepChem [40] TextCNN method (https://github.com/deepchem).

Each convolution had a kernel size from the list (1, 2, 3, 4, 5, 6, 7, 8, 9, 10, 15, 20) and produced the following number of filters (100, 200, 200, 200, 200, 100, 100, 100, 100, 100, 160, 160), respectively. After a GlobalMaxPool operation and the subsequent concatenation of the pooling results, the data went through Dropout [41] (rate = 0.25), Dense (N = 512), Highway [42] layers, and, finally, converted to the output layer which consisted of only one neuron for regression and two neurons for classification tasks. The weights of the Transformer’s part were frozen in all experiments. All models used the Adam optimizer with Mean Squared Error or Binary Cross-Entropy loss depending on the problem at hand. A fixed learning rate λ = 10^–4^ was used. Early-stopping was used to prevent overfitting, to select a best model, and to reduce training time. OCHEM calculations were performed using canonical SMILES as well as ten-fold augmented SMILES during both training and prognosis. This number of SMILES augmentations was found to be an optimal one in our previous study [43]. An average value of the individual predictions for different representation of the same molecule was used as the final model prediction to calculate statistical parameters.

The same five-fold cross-validation procedure was used to compare the models with the results of our previous study [43]. The coefficients of determination [44]

1 $$r^2 = 1 - SS_\text{res}/SS_\text{tot}$$

where SS_tot_ is total variance of data and SS_res_ is residual unexplained variance of data was used to compare regression models and Area Under the Curve (AUC) was used for classification tasks.

#### Validation datasets

We used the same datasets (9 for regression and 9 for classification) that were exploited in our previous studies [11, 22]. Short information about these sets as well as links to original works are provided in Table 1. The datasets are available on the OCHEM environment on https://ochem.eu.

**Table 1:** Descriptions of datasets used in the work

Code	Description	Size	Code	Description	Size
Regression tasks			Classification tasks		
MP	Melting point [45]	19,104	HIV	Inhibition of HIV replication [46]	41,127
BP	Boiling point [47]	11,893	AMES	Mutagenicity [48]	6542
BCF	Bioconcentration factor [47]	378	BACE	Human β-secretase 1 (BACE-1) inhibitors [46]	1513
FreeSolv	Free solvation energy [46]	642	Clintox	Clinical trial toxicity [46]	1478
LogS	Solubility [49]	1311	Tox21	In-vitro toxicity [46]	7831
Lipo	Lipophilicity [50]	4200	BBBP	Blood–brain barrier [46]	2,039
BACE	IC50 of human β-secretase 1 (BACE-1) inhibitors [46]	1513	JAK3	Janus kinase 3 inhibitor [51]	886
DHFR	Dihydrofolate reductase inhibition [52]	739	BioDeg	Biodegradability [53]	1737
LEL	Lowest effect level [54]	483	RP AR	Endocrine disruptors [55]	930

## Results and discussion

### SMILES canonicalization model

**Fig. 5:**
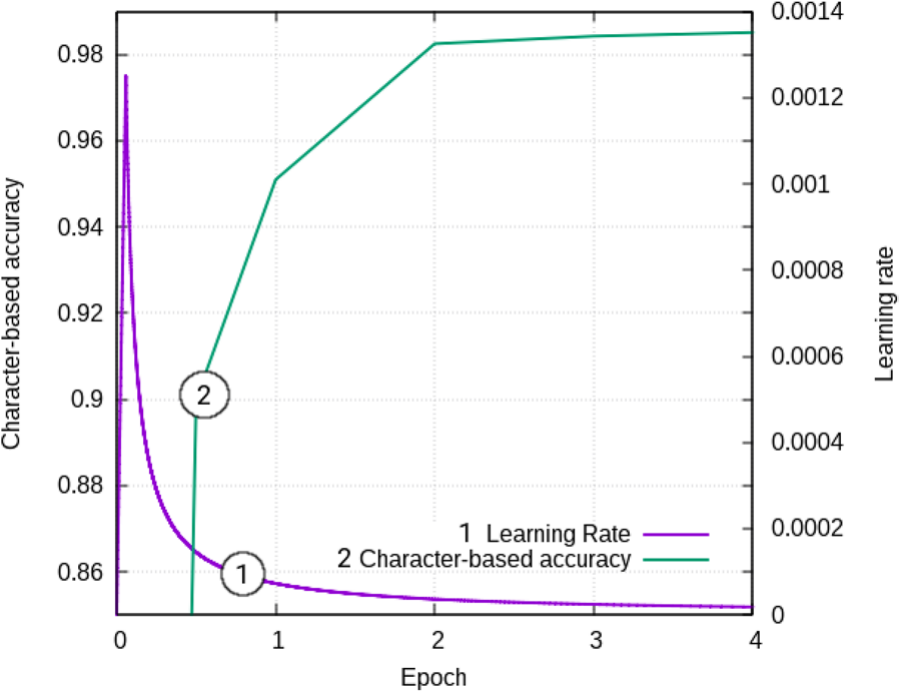
Learning curves: 1) learning rate schedule (axes bottom and right), and 2) character-based accuracy (axes bottom and left) on the training dataset for the first four epochs

The Transformer model was trained for 10 epochs with the learning rate changing according to the formula:

2 $$\lambda =factor * min\,(1.0, step/ warmup) / max\,(step, warmup)$$

where *factor* = 20, *warmup* = 16,000 steps, and if λ < 10^–4^ then λ = 10^−4^. The settings for the learning rate were similar to those used in our retro-synthesis study. Each epoch contained 275,907 steps (batches). No early-stopping or weight-averaging was applied. Learning curves are shown in Fig. 5.

To validate the model, we sampled 500,000 ChEMBL-like SMILES (only 8,617 (1.7%) of them were canonical) from a generator [56] and checked how accurately the model can restore canonical SMILES for these molecules. We intentionally selected the generated SMILES keeping in mind possible applications of the proposed method in artificial intelligence-driven pipelines of *de-novo* drug development. The model correctly canonicalized 83.6% of all samples, Table 2.

**Table 2:** Validation of canonicalization model

Strings	All	Correctly canonicalized
All	500,000	418,233 (83.6%)
Stereo (with @)	77,472	28,821 (37.2%)
Cis/trans (with / or \)	54,727	40,483 (73.9%)

### QSAR modeling

**Fig. 6:**
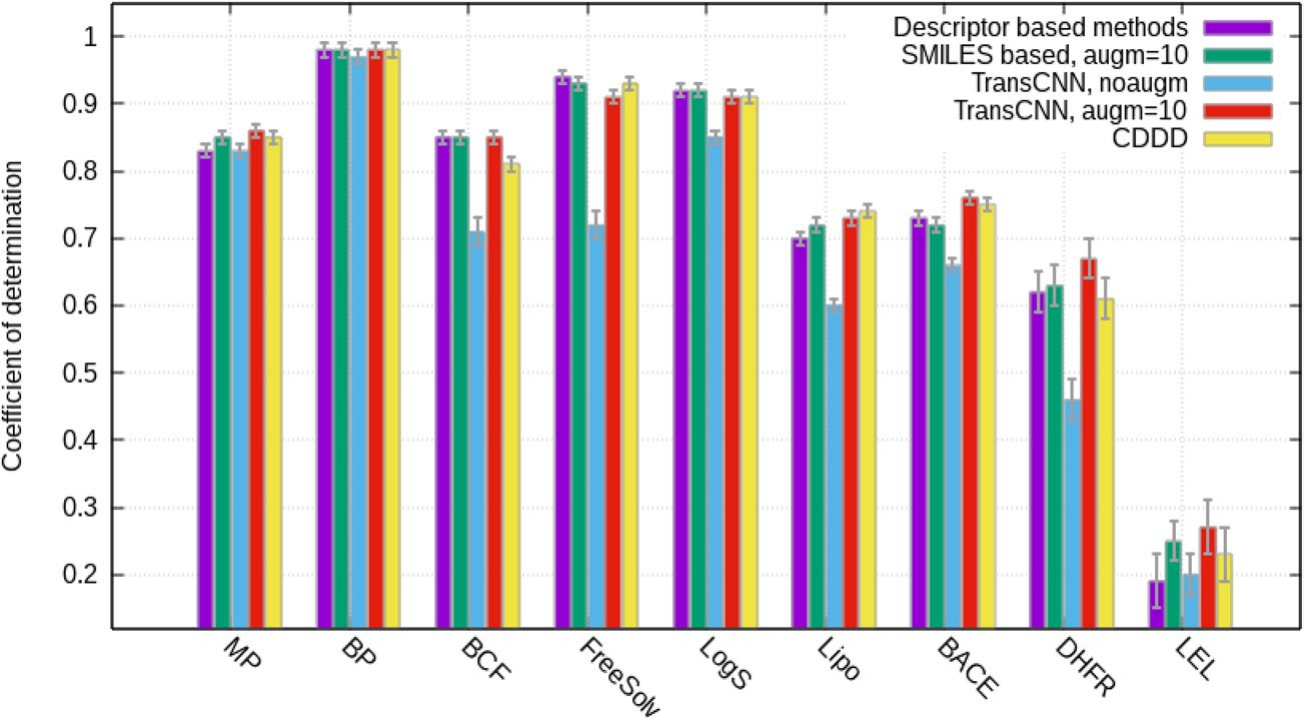
Coefficient of determination, r^2^, calculated for regression sets (higher values are better)

**Fig.7:**
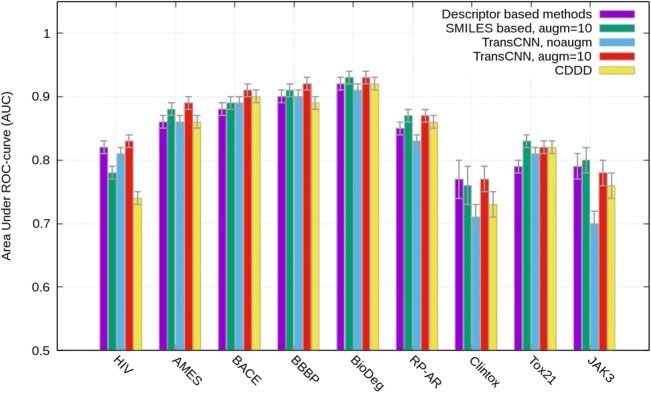
AUC calculated for classification sets (higher values are better)

For the QSAR modelling the saved embedding was used. The training was done using a fixed learning rate λ = 0.001 for n = 100 epochs. Early stopping with 10% randomly selected SMILES was used to identify the optimal model. Table 3, Fig. 6 compares results for regression datasets while Table 4, Fig. 7 compares classification tasks. The standard mean errors of the values were calculated using a bootstrap procedure as explained elsewhere [53].

**Table 3:** Coefficient of determination, r^2^, calculated for regression sets (higher values are better)

Dataset	Descriptor based methods^2^	SMILES based (augm = 10)^a^	Transformer-CNN, no augm	Transformer-CNN, augm = 10	CDDD descriptors^b^
MP	0.83	0.85	0.83	0.86	0.85
BP	0.98	0.98	0.97	0.98	0.98
BCF	0.85	0.85	0.71 ± 0.02	0.85	0.81
FreeSolv	0.94	0.93	0.72 ± 0.02	0.91	0.93
LogS	0.92	0.92	0.85	0.91	0.91
Lipo	0.7	0.72	0.6	0.73	0.74
BACE	0.73	0.72	0.66	0.76	0.75
DHFR	0.62 ± 0.03	0.63 ± 0.03	0.46 ± 0.03	0.67 ± 0.03	0.61 ± 0.03
LEL	0.19 ± 0.04	0.25 ± 0.03	0.2 ± 0.03	0.27 ± 0.04	0.23 ± 0.04

We omitted the standard mean errors, which are 0.01 or less, for the reported values

^a^Results from our previous study [22]. ^b^Best performance calculated with CDDD descriptors obtained using autoencoder Sml2canSml from [27]

**Table 4:** AUC calculated for classification sets (higher values are better)

Dataset	Descriptor based methods^a^	SMILES based (augm = 10)^2^	Transformer-CNN, no augm	Transformer-CNN, augm = 10	CDDD descriptors^b^
HIV	0.82	0.78	0.81	0.83	0.74
AMES	0.86	0.88	0.86	0.89	0.86
BACE	0.88	0.89	0.89	0.91	0.9
Clintox	0.77 ± 0.03	0.76 ± 0.03	0.71 ± 0.02	0.77 ± 0.02	0.73 ± 0.02
Tox21	0.79	0.83	0.81	0.82	0.82
BBBP	0.90	0.91	0.9	0.92	0.89
JAK3	0.79 ± 0.02	0.8 ± 0.02	0.70 ± 0.02	0.78 ± 0.02	0.76 ± 0.02
BioDeg	0.92	0.93	0.91	0.93	0.92
RP AR	0.85	0.87	0.83	0.87	0.86

We omitted the standard mean errors, which are 0.01 or less, for the reported values

^a^Results from our previous study [22]. ^b^Best performance calculated with CDDD descriptors obtained using Sml2canSml autoencoder from [27]

With an exception of a few datasets, the proposed method provided similar or better results than those calculated using descriptor-based approaches as well as the other SMILES-based approaches investigated in our previous study [43]. The data augmentation was critically important for the Transformer-CNN method to achieve its high performance. We used augmentation n = 10, i.e., 10 SMILES were randomly generated and used for model development and application, which was found optimal in the aforementioned previous study.

Similar to Transformer-CNN the Sml2canSml used an internal representation, which was developed by mapping arbitrary SMILES to canonical SMILES. The difference was that Sml2canSml generated a fixed set of 512 latent variables (CDDD descriptors), while the Transformer-CNN representation had about the same length as the initial SMILES. Sml2canSml CDDD could be used as descriptors for any traditional machine learning methods while Transformer-CNN required convolutional neural networks to process the variable length output and to correlate it with the analysed properties. Sml2canSml was added as CDDD descriptors to OCHEM. These descriptors were analysed by the same methods as used in the previous work, i.e., LibSVM [57], Random Forest [58], XGBoost [59] as well as by Associative Neural Networks (ASNN) [60] and Deep Neural Networks [61]. Exactly the same protocol, fivefold cross-validation, was used for all calculations. The best performance using the CDDD descriptors was obtained by ASNN and LibSVM methods, which contributed models with the highest accuracy for seven and five datasets respectively (LibSVM method provided the best performance in the original study). Transformer-CNN provided better or similar results compared to the CDDD descriptors for all datasets with an exception of Lipo and FreeSolv. It should be also mentioned that CDDD descriptors could only process molecules which satisfy the following conditions:

logP ∈ (−5,7) and

mol_weight ∈ (12,600) and

num_heavy_atoms ∈ (3, 50) and

molecule is organic.

These limitations appeared due to the preparation of the training set to develop the Sml2canSml encoder. The limitations resulted in the exclusion of a number of molecules, which failed one or several of the above conditions. Contrary to the Sml2canSml encoder, we trained Transformer-CNN with very diverse molecules from ChEMBL and thus the developed models could be applied to any molecule which can be processed by RDKiT. The exclusion of molecules for which CDDD descriptors failed to be calculated did not significantly change the results of Transformer models: some models improved while others decreased their accuracy for ~ 0.01 respective performance values. For example, for Lipo and FreeSolv sets the accuracy of the Transformer-CNN model increased to r^2^ = 0.92 and 0.75 respectively, while for BBB the AUC decreased to 0.91.

### Interpretability of the model

Layer-wise relevance propagation was used to interpret the models. For gated connections (in HighWay block) we implemented the signal-take-all redistribution rule [62] while all other Dense and Convolutional layers were well fitted in the LRP framework [32] without any adaptation. In this work, we stopped the relevance propagation on the output of the Transformer’s encoder which is position-wise. It should be noted that we froze the encoder part of the network during QSAR model training. Summing up all the individual features for each position in the SMILES string calculated its contribution to the final result. If the LRP indicated a reasonable explanation of the contributions of fragments then one can trust that the model made predictions based on detected fundamental structure–property relationships. For explanation we selected classification (AMES mutagenicity) and regression (water solubility) models.

### AMES mutagenicity

The AMES test is a widely used qualitative test to determine the mutagenic potential of a molecule, from which extensive structural alerts collections were derived [63]. Examples of these alerts are aromatic nitros, N-oxides, aldehydes, monohaloalkenes, quinones, etc. A QSAR model for AMES had to pay special attention to these and similar groups to be interpretable and reliable. The Transformer-CNN model built on 6542 endpoints (3516 mutagenic and 3026 nonmutagenic) results in AUC = 0.89, Table 4.

The structure of *1-Bromo-4-nitrobenzene* gave the positive AMES test. The output of the LRP procedure for one of possible SMILES for this compound, namely 1c([N +]([O-]) = O)ccc(c1)Br, is shown in Table 5.

**Table 5:** Local relevance conservation for c1c([N +]([O−]) = O)ccc(Br)c1

Layer	Relevance, R (L + 1)	Relevance, R (L)	Delta, R (L + 1)-R (L)	Bias, Delta / R (L + 1) *100%
Result	0.98119	–	–	–
HighWay Output	0.98119	0.9300	0.0512	5.21
HighWay Input	0.9300	0.7227	0.2073	22.3
DeMaxPool	0.7227	0.7371	− 0.0144	− 1.98
Conv1	0.0090	0.0117	− 0.00271	− 30.1
Conv2	0.1627	0.1627	0^a^	0
Conv3	− 0.0443	− 0.0443	0	0
Conv4	0.0191	0.0191	0	0
Conv5	− 0.0984	− 0.0984	0	0
Conv6	− 0.0136	− 0.0136	0	0
Conv7	0.0806	0.0806	0	0
Conv8	0.0957	0.0957	0	0
Conv9	0.1528	0.1528	0	0
Conv10	0.0845	0.0845	0	0
Conv15	0.1038	0.1038	0	0
Conv20	0.1851	0.1851	0	0
Total	0.98119	0.7398	0.2414	24.6

^a^All 0 values were all less than 10^− 5^

According to the LRP, the relevance was constant during the propagation:

3 $$y=R =f(x)={\sum }_{l\in (L)}{R}_{l} = {\sum }_{l\in (L-1)}{R}_{l} ={\sum }_{l\in (L-2)}{R}_{l} = {\sum }_{l\in (1)}{R}_{l} .$$

Here (L) stood for a set of neurons in the last layer, (L−1)—in the layer before the last layer, and so on. Each layer in the Transformer-CNN network contained biases (B), and thus some relevance dissipated on them. Therefore the above equation was corrected to:

4 $${\sum }_{l\in (L)}{R}_{l} = {\sum }_{l\in \left(L-1\right)}{R}_{l} + B.$$

We calculated how much of the relevance was taken by biases and reported these values in the output of the ochem.py script. Table 5 clearly shows that 24.6% of the output signal was taken by biases and 75.4% were successfully propagated to position-wise layers, which we used to interpret the model. If less than 50% of the signal came to the input, it may indicate an applicability domain problem or technical issues with relevance propagation. In these cases the interpretation could be questioned.

**Fig. 8:**
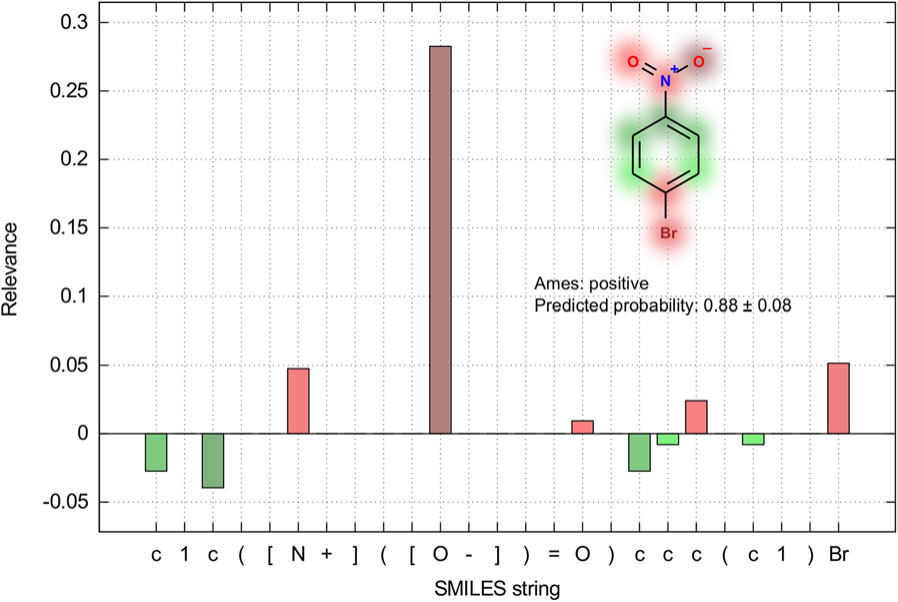
Visualization of atom contributions, in the case of a mutagenic compound. The red color stands for mutagenic alerts, color green against it

Iterating through all non-hydrogen atoms, the interpretation algorithm picked up an atom and drew a SMILES from it. Thus, every molecule had a corresponding set of SMILES equal to the number of atoms. The LRP was used for every SMILES, and then the individual predictions were averaged for the final output. 1-Bromo-4-nitrobenzene was predicted as mutagenic with the score 0.88. Impacts of the atoms on the property is depicted in Fig. 8. The model predicted this compound as mutagenic because of the presence of nitro and halogen benzene moieties. Both are known to be structural alerts for mutagenicity [63]. Charged oxygen provided a bigger impact than the double bonded one in the nitro group because its presence contributed to the mutagenicity for nitro and also for N-oxide compounds.

### Aqueous solubility

Solubility is a crucial property in drug-development. To have a fast, robust, and explainable tool for its prediction and interpretation is highly desirable by both academia and industry. The Transformer-CNN model built on 1311 compounds had the following statistics: *q*^*2*^ = 0.92 and *RMSE*_*p*_ = 0.57 [64]. For demonstration of its interpretability we choose haloperidol—a well-known antipsychotic drug with 14 mg/l water solubility [65].

**Fig. 9:**
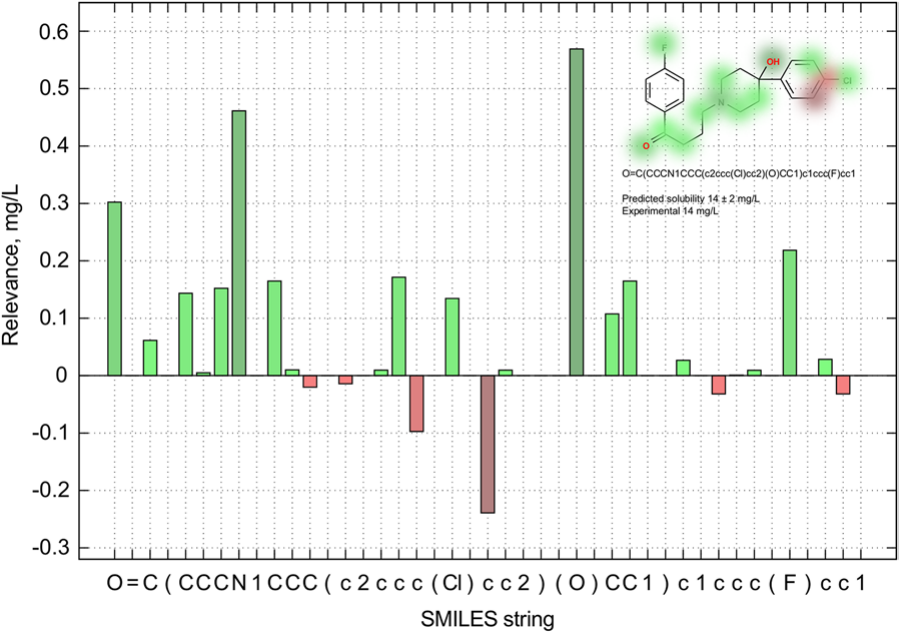
Visualization of atom contributions to aqueous solubility of haloperidol. The greep bars stand for more soluble features, whereas the red ones show the opposite effect

The Transformer model calculated the same solubility 14 ± 2 mg/L for this compound. The individual atom contributions are shown in Fig. 9. Hydroxyl, carbonyl, aliphatic nitrogen, and halogens contributed mostly to the solubility. These groups can form ionizable zones in the molecule thus helping water to dissolve the substance. Several aromatic carbons had negative contributions, which was expected since aromatic compounds are poorly soluble in water. Thus the overall explanation made sense, and the model had an excellent statistics not because of spurious correlations, but because it found the right fragmental features responsible for modelled property. The standalone program contributed in this work has no dependencies on machine learning frameworks, it is easy to install, to use, and to interpret the modelling results. This will make it an indispensable work-horse for drug-development projects world-wide.

## Conclusions and outlook

For the first time we proposed a SMILES canonicalization method based on Transformer architecture that extracts information-rich real-value embeddings during the encoding process and exposes them for further QSAR studies. Also, for the first time we developed a framework for the interpretation of models based on the Transformer architecture using a layer-wise relevance propagation (LPR) approach.

TextCNN approaches efficiently worked with embeddings generated by Transformer, and the final quality of the QSAR models was higher compared to the models obtained with the state-of-the-art methods on the majority of diverse benchmark datasets. The Transformer-CNN architecture required less than a hundred iterations to converge for QSAR tasks to model various biological activity or physico-chemical properties. It can be easily embedded into *de-novo* drug development pipelines. The model predictions interpreted in a fragment contribution manner using the LPR could be useful to design new molecules with desired biological activity and ADMETox properties. The source code is available on https://github.com/bigchem/transformer-cnn as well as an on-line version on https://ochem.eu. For solubility and AMES mutagenicity we also deposited standalone models in the GitHub repository, which not only predict the respective properties but also provide interpretations of predictions.

The Transformer-CNN predicts the endpoint based on an average of individual prognosis for a batch of augmented SMILES belonging to the same molecule. The deviation within the batch can serve as a measure of a confidence interval of the prognosis. Dissipation of relevance on biases as well as analysis of restored SMILES can be used to derive the applicability domains of models. These questions will be addressed in the upcoming studies.

Also, as a comment, we do not think that the authors benchmarking their methods are impassioned about their work. Such benchmarking could be properly done by other users, and we do hope to see the proposed method used soon in future publications. But indeed, remarkably, in this work we saw an outstanding performance of the proposed architecture, which provided systematically better or at least similar results compared to the best descriptor-based approaches as well as several analysed deep neural network architectures. Even more remarkably, the Transformer CNN has practically no adjustable meta parameters and thus does not require spending time to tune hyperparameters of neural architectures, use the grid search to optimise Support Vector Machines, optimise multiple parameters of XGBoost, apply various descriptors filtering and preprocessing, which could easily contribute to the overfitting of models. This as well as the possibility to interpret models makes Transformer CNN a Swiss-knife for QSAR modeling and interpretation, which will help to make the QSAR great again!

## Data Availability

The source code of the Transformer-CNN is available on https://github.com/bigchem/transformer-cnn. Ready-to-use implementation, training datasets, and models are available on OCHEM https://ochem.eu.

## Abbreviations

ADMETox: Absorption, distribution, metabolism, excretion and toxicity
ANN: Artificial neural network
CNN: Convolutional neural network
LSTM: Long Short-Term memory
OCHEM: On-line chemical database and modeling environment
SMILES: Simplified Molecular-Input Line-Entry System
QSAR/QSPR: Quantitative Structure Activity/Property Relationship
RF: Receptive field
RNN: Recurrent Neural Network
CNN: Convolutional Neural Network
Transformer-CNN: Transformer Convolutional Neural Network
